# Complementary Therapies for Idiopathic Hirsutism: Topical Licorice as Promising Option

**DOI:** 10.1155/2015/659041

**Published:** 2015-07-27

**Authors:** Gita Faghihi, Fariba Iraji, Bahareh Abtahi-Naeini, Bahar Saffar, Ali Saffaei, Mohsen Pourazizi, Abolfazl Aslani, Mohammad Ali Nilforoushzadeh

**Affiliations:** ^1^Skin Diseases and Leishmaniasis Research Center, Department of Dermatology, Isfahan University of Medical Sciences, Isfahan, Iran; ^2^Skin and Stem Cell Research Center, Tehran University of Medical Sciences, Tehran, Iran; ^3^Department of Pathology, Isfahan University of Medical Sciences, Isfahan, Iran; ^4^Pharmacy Students' Research Committee, School of Pharmacy, Isfahan University of Medical Sciences, Isfahan, Iran; ^5^Cancer Research Center, Semnan University of Medical Sciences, Semnan, Iran; ^6^Students' Research Committee, Isfahan University of Medical Sciences, Isfahan, Iran; ^7^School of Pharmacy and Novel Drug Delivery Systems Research Center, Isfahan University of Medical Sciences, Isfahan, Iran

## Abstract

Hirsutism is one of the most prevalent health problems in women. The aim of the study was to compare the effect of 755 nm alexandrite hair removal laser with that of alexandrite laser plus topical licorice on the improvement of idiopathic hirsutism. A double-blind, randomized placebo-controlled study was performed on 90 female subjects. The patients were divided into two groups: alexandrite laser plus 15% licorice gel (group A) and placebo (group B). Each subject received one of both products over one side of the face, twice daily for 24 weeks on the hirsute locations. Each group underwent five sessions of alexandrite laser at 6-week intervals. To minimize the effects of confounding variables, the test was performed on two separate zones of patients' skin. The mean ± SD numbers of terminal hairs in group A were 7.05 ± 4.55 for zone 1 and 6.06 ± 3.70 for zone 2. In group B, they were 3.18 ± 1.75 for zone 1 and 2.49 ± 1.63 for zone 2. The difference in the mean number of terminal hairs was statistically significant between the two groups (*p* < 0.001), and there were no serious adverse reactions. The treatment of idiopathic hirsutism with 755 nm alexandrite laser plus topical licorice is more effective than alexandrite laser only.

## 1. Introduction

Hirsutism is defined as the presence of excessive terminal (coarse) hair in androgen-sensitive areas of the body [1]. It is also one of the most prevalent health problems in women, with a prevalence of about 10%, and can significantly and negatively impact their quality of life [2].

Licorice or* Glycyrrhiza glabra* has been used as a medicinal plant since ancient times [3]. The hydrolysis of glycyrrhizic acid, an active component of licorice, produces two molecules of the d-glucuronic acid and aglycone 18*β*-glycyrrhetinic acid, which is responsible for most of the metabolic effects (agonist of mineralocorticoid receptors and mild inhibitor of androgen synthesis) and the estrogen-like activity of licorice [3, 4].

In recent years, there has been an increasing emphasis on the potential for alternative plant-derived antiandrogen compounds, which reflects the fact that the medications used in clinical practice are not much effective for the majority of patients. Thus, there is clearly a need for more efficacious and greater variety of drugs to treat androgen-related disorders such as hirsutism [5].

The aim of the study was to compare the effect of 755 nm alexandrite hair removal laser with that of alexandrite laser plus topical licorice (agonist of mineralocorticoid receptors and mild inhibitor of androgen synthesis) on clinical improvement of mild to moderate idiopathic hirsutism.

## 2. Patients and Methods

### 2.1. Study Design and Participants

**Figure 1:**
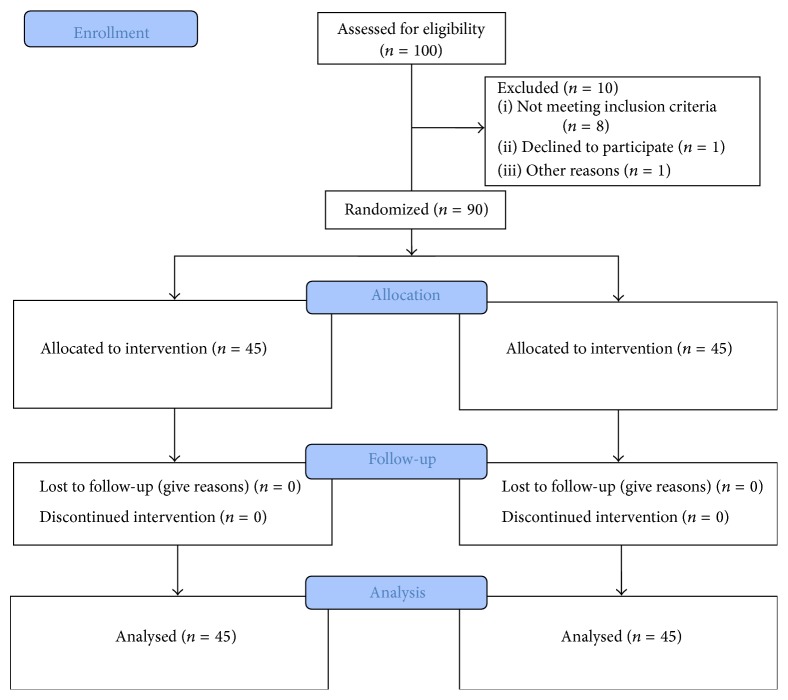
CONSORT flow diagram of the study.

A double-blind, randomized placebo-controlled study was done to assess the efficacy of a 5-session alexandrite laser treatment and 24-week topical 15% licorice gel treatment on mild to moderate idiopathic hirsutism. The COSORT flow diagram of this study is shown in Figure 1.

This study was approved by the Ethics Committee of Isfahan University of Medical Sciences, Isfahan, Iran (Research Project number 388125). 45 of subjects were Patients who aged 15 to 45 years and the skin phototype of patients were II to IV. They were suffering of mild to moderate idiopathic hirsutism on both sides of the face (upper lip, cheek, and chin). Modified Ferriman-Gallwey scale was used for case selection [6]. Exclusion criteria were as follows: pregnancy or intention to become pregnant; breastfeeding; severe systemic diseases; increased serum androgen level, irregular menstrual cycle history of androgens or antiandrogen, and oral contraceptive agents intake within the previous 2 months; and allergy or hypersensitivity to any components of the product under investigation.

After written informed consent, the patients were divided into two randomization groups according to sequential entering into two matched blocks for 15–30 and 31–45 years of age.

Alexandrite laser plus 15% licorice gel (*n* = 45) (group designated as A) and alexandrite laser plus placebo (*n* = 45) (group designated as B): at the first visit, a general medical history and thorough clinical examination were performed. Questionnaires including information about age, sex, and skin photo type were completed.

### 2.2. Study Protocol

The product to be tested was 15% licorice gel packaged in tube as the placebo packaged in identical tube. Only the pharmacy technician who was responsible for dispersing the tubes was aware. No other member of the team was aware of the contents of the dispersed tubes.

In a running period of 48 hours, gel was applied to a small area of the forearm skin for detection of any side effects.

Each subject received one of both products with the dose of half tip finger unit over one side of face, twice daily for 24 weeks on the hirsute locations. Each group underwent five treatment sessions at 6-week intervals of alexandrite laser (755 nm alexandrite laser GentleLASE, Candela Co., USA). The treatment settings were according to skin phototype of the skin: fluence: 14–20 millijoules and pulse width: 35–40 milliseconds.

Patients were also instructed to avoid the use of any systemic or topical drugs during the course of treatment.

The primary outcome was the changes in terminal hair density. The terminal hair was counted by the investigator using manual magnification on the treatment and control sites at baseline and on each follow-up visit. The counting was assessed by marking each counted hair with a pen to ensure that each hair was only registered once.

To minimize the effects of confounding variables, the test is performed on two separate zones of patients' skin (in center and periphery of hirsute area of the face). The results are illustrated in graphs for easy comparison.

Photographs were taken using identical camera settings (Canon D30, Canon Inc., Tokyo, Japan), lighting, and patient positioning at baseline and after the last treatment session. Local tolerance was evaluated at each assessment period (6-week intervals) by direct evaluation. Also, investigators assessed and recorded possible side effects, including pruritus, burning, and contact dermatitis, at each visit.

### 2.3. Preparation of the Formulations

Licorice gel was prepared from licorice (*Glycyrrhiza glabra*) root powder extract (produced locally in southern parts of Iran (Arsanjan Factory, Fars Province, Iran)) that had been soaked in ethanol 80% and then mixed with lubricant nonallergenic gel (sodium carboxymethylcellulose) up to 15% concentration. Final formulations for clinical trial were controlled microbiologically based on USP XXIV (USP 24) [7]. The vehicle gel formulation was used as placebo. The licorice gel and vehicle neutral gel were filled in similar tubes that were marked as “A” or “B.”

### 2.4. Statistical Analysis

Statistical evaluation was done using SPSS for Windows version 18.0 (SPSS Inc., II, USA). Changes in the numbers of terminal hairs between two groups were compared by Student's *t*-test and statistical significance was defined as *p* value < 0.05.

## 3. Results

A total of 90 hirsute patients were included in this study. All patients (100%) completed the study. The difference in mean ages of the two groups was not statistically significant (*p* = 0.76). Also, there was no significant difference between skin phototypes in the two groups (*p* = 0.73).

Statistical Student's test-*t* showed that there is no significant difference between the two zones which implies the minimal effects of confounding variables (*p* > 0.05).

**Figure 2:**
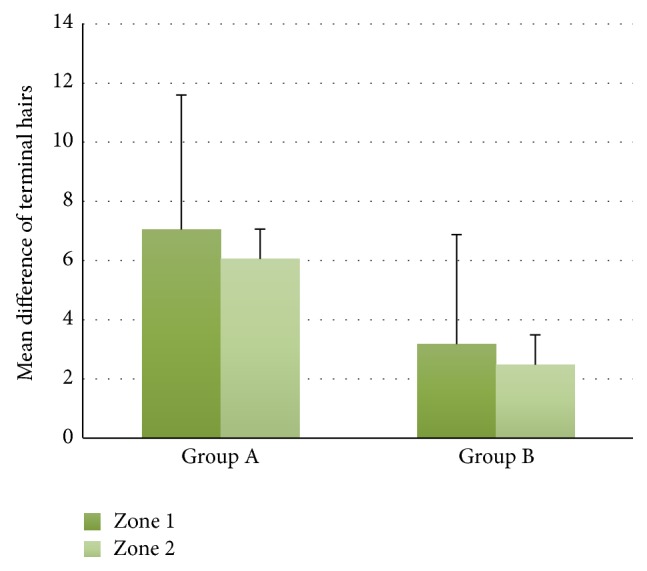
The mean ± SD difference of terminal hairs between groups (*p* < 0.001).

The mean ± SD difference of terminal hairs in group A was 7.05 ± 4.55 for zone 1 and 6.06 ± 3.70 for zone 2 consecutively. Conversely in group B, it was 3.18 ± 1.75 for zone 1 and 2.49 ± 1.63 for zone 2 (Figure 2). Statistical Levene's test shows significant difference between those two groups (*p* < 0.001).

In patients who were in group A and had skin type II the mean difference of terminal hair in zone 1 was 7.63 ± 1.28 and 6.11 ± 0.99 in zone 2, in skin type III it was 5.30 ± 0.61 for zone 1 and 5.65 ± 0.82 for zone 2, and finally in skin type IV it was 7.94 ± 1.15 for zone 1 and 7.05 ± 1.06 for zone 2 consecutively. In group B the mean difference of terminal hair, in patients with skin type II, was 2.66 ± 1.48 for zone 1 and 2.21 ± 1.55 for zone 2, in patients with skin type III, it was 3.59 ± 1.86 for zone 1 and 2.73 ± 1.82 for zone 2, and also, in patients with skin type IV, it was 2.66 ± 0.57 for zone 1 and 2.66 ± 0.33 for zone 2. The above-mentioned results are summarized in Table 1.

**Figure 3:**
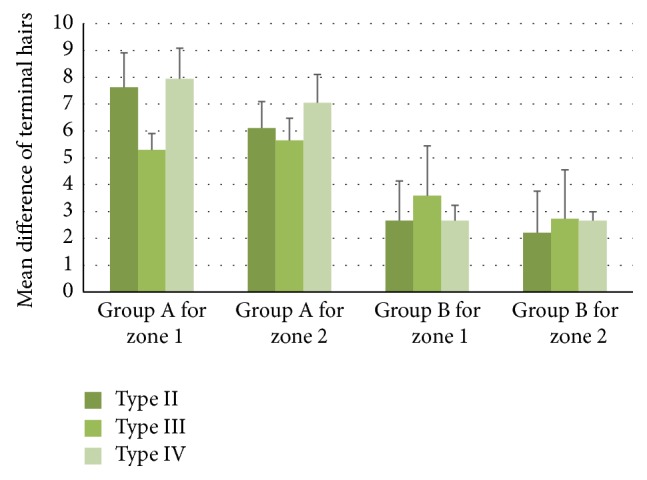
The comparison of mean difference of terminal hairs between skin types.

Using Levene's test, there was no tendency toward higher improvement with darker skin phototype and this was not statistically significant (*p* > 0.05) (Figure 3).

**Figure 4:**
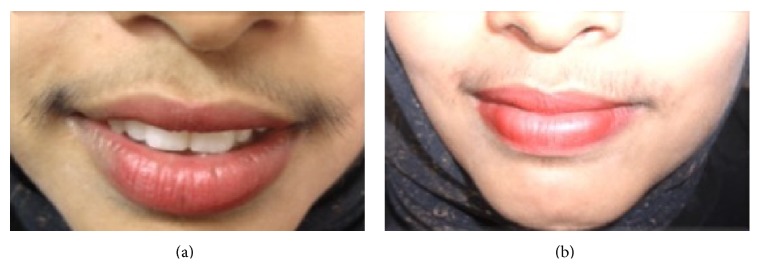
(a) The patient with hirsutism before treatment and (b) the same patient after treatment with laser and licorice gel.

There were no serious adverse reactions; also we did not observe any case of contact dermatitis or hypersensitivity reaction to topical product throughout the follow-up period. All patients tolerated the laser treatments well, and no long-term adverse effects were observed and no patient gave up the study due to side-effects in either group (Figure 4).

## 4. Discussion

The results of the present study reveal that the treatment of idiopathic hirsutism with 755 nm alexandrite laser plus topical licorice is more effective than alexandrite laser only. To the best of our knowledge, precisely randomized, placebo-controlled study on the effect of licorice on hirsutism has not been performed as of now.

Hirsutism reflects the interaction among the circulating and local androgen concentrations and the sensitivity of hair follicle to androgens [8]. It is thought that idiopathic hirsutism results from excessive sensitivity of the skin to normal levels of circulating androgens [9].

Hirsutism should be treated using combination therapy, including androgen suppression, peripheral androgen blockade, mechanical/cosmetic amelioration, and removal of unwanted hairs [10].

Laser-assisted hair removal is a well-tolerated and effective technique for patients who desire permanent reduction of hair growth. The ideal patient for laser hair removal is one with light skin with black coarse hair [10, 11].

Physical methods of removing hair can be effective and their use is reasonable either alone or as a supplement to drug therapy [11].

The Cosmetic Ingredient Review (CIR) Expert Panel noted that the ingredients used in this safety assessment are not plant extracts, powders, or juices but are rather specific chemical species that may be isolated from the licorice plant. Although these chemicals may be isolated from plant sources, steps should be taken to ensure that pesticide and toxic metal residues are below acceptable levels [12]. Nevertheless, topical licorice could be used in the treatment of hirsutism as a complementary option.

Few studies have shown that licorice had a mild inhibitory effect and an estrogen-like activity on androgen synthesis [3, 4]. Different mechanisms could be explained for this phenomenon; licorice can affect androgen metabolism. Studies have stated that licorice blocks the activity of 17-hydroxysteroid dehydrogenase (17HSD) and 17,20-lyase, stimulates the activity of aromatase [13, 14], and also affects 5*α*- and 5*β*-reductase activity [15]. All of these enzymes are involved in the synthesis and/or metabolism of androgens and estrogens. Licorice has also been used for the treatment of sterility in women [3]. Yaginuma et al. showed the effect of herbal product containing glycyrrhizic acid on the reduction of serum testosterone and induction of regular ovulation and consequently licorice was proposed for the treatment of hirsutism and polycystic ovary syndrome [13]. Moreover, the metabolites of licorice, glabridin, and glabrene have an estrogen-like effect and have been used for the treatment of menopause [16]. In addition, Armanini et al. reported a significant reduction in serum testosterone in healthy women and men over 2 months of licorice intake [17, 18]. The root of licorice plant is rich in triterpenes especially glycyrrhizin, which has a beneficial effect on estrogen receptors [4, 19]. On the other hand, in a recent study, Josephs et al. found normal values of salivary testosterone with ingestion of licorice [20]. Another study showed the positive effects of glycyrrhizin and glycyrrhizic acid, which are a component of the root extract of licorice, in reducing unwanted hairs. This reduction is possibly due to the enzymatic effect on melatonin generation cycle [12]. An earlier in vivo study explained that this reduction is probably due to inhibition of tyrosinase activity [21].

Our study showed results specific to skin types, which could be because of the fact that blacker the skin, the higher the amount of melatonin. This increased amount of melatonin ultimately leads to a better response to the current therapies, on the basis of which we conclude that the role of skin type in hirsutism treatment is undeniable [22]. However, this study shows no meaningful differences in skin types in terms of treatment response and the results are in accordance with those of Azziz et al. who reported that the skin type has no important difference in hirsutism treatment [22]. Further studies are necessary to clarify this controversy.

In addition, mechanisms other than the presence of melatonin should also be considered. For example, Essah et al. showed that those regions of skin, such as the upper lip, producing more androgen respond to hirsutism treatment in a different manner [23]. However, our results indicate no significant difference between the two skin zones in the patients.

Therefore, this study concludes that the effect of licorice may be different from that of only androgenic effect of root, and hence further study is needed for a better understanding of licorice mechanism.

## 5. Conclusion

The addition of licorice to hair removal laser may be more effective than hair removal laser only, and, in cases requiring hair removal laser for the treatment of hirsutism, better outcome could be achieved if the physician combines it with topical licorice as an adjuvant treatment. Further studies are required for a better elucidation of the clinical implications of these data from clinical and/or biochemical perspectives of hirsutism.

## Figures and Tables

**Table 1:** The mean ± SD difference of terminal hairs in group A and group B regarding skin phototype.

Groups	Skin phototype	Patients	Zone 1	Zone 2	*p* value
Group A	II	20	7.63 ± 1.28	6.11 ± 0.99	0.243 in zone 1 and 0.673 in zone 2
	III	15	5.30 ± 0.61	5.65 ± 0.82	
	IV	10	7.94 ± 1.15	7.05 ± 1.06	

Group B	II	21	2.66 ± 1.48	2.21 ± 1.55	0.182 in zone 1 and 0.585 in zone 2
	III	21	3.59 ± 1.86	2.73 ± 1.82	
	IV	3	2.66 ± 0.57	2.66 ± 0.33	
